# Primary breast angiosarcoma in a postmenopausal woman: A case report

**DOI:** 10.1016/j.ijscr.2023.108700

**Published:** 2023-08-19

**Authors:** Callie Killoran, Thushara Dissanayake

**Affiliations:** a Department of Surgery, Sunshine Coast University Hospital, 6 Doherty Street, Birtinya, Queensland 4575, Australia; b Griffith University, School of Medicine and Dentistry, Parklands Drive, Southport, Gold Coast Campus Griffith University, Queensland 4222, Australia

**Keywords:** Primary angiosarcoma, Breast angiosarcoma, Rare breast cancer, Biopsy, Diagnosis, Case report

## Abstract

**Introduction and importance:**

Primary angiosarcoma of the breast is a rare malignancy with an associated poor prognosis due to its high rates of reoccurrence and metastasis It is most common in females presenting in their 3rd and 4th decades with no evidence of hormonal dependency. Cases usually present with rapidly progressing non-tender breast lump.

**Case presentation:**

A 75-year-old female referred for triple assessment of a rapidly enlarging right breast lump. The patient underwent multiple investigations for work-up and to aid in the diagnosis of a moderately differentiated angiosarcoma of the breast.

**Clinical discussion:**

Diagnostic dilemmas remain due to the non-specific findings on standard radiological investigations and high false-negative results on core-biopsy. Consideration should be made in those with a high clinical suspicion for magnetic resonance imaging and excisional biopsy. Although limited research, first line management of those without metastatic disease is radical surgery.

**Conclusion:**

Breast primary angiosarcoma is a rare entity in post-menopausal women that should be considered in the differentials of breast lumps. Standardized information is limited though current management includes local control with radical surgery. The role for neoadjuvant therapy, adjuvant radiotherapy or chemotherapy is still unclear.

## Highlights

• Highly aggressive malignant soft tissue tumour.
• Primary breast angiosarcoma less common than secondary breast angiosarcoma.
• Rare condition amongst breast lesions.
• No evidence to support hormone dependency.
• Current mainstay treatment is debulking surgery.

## 1 Introduction

Angiosarcoma (AS) is a rare soft tissue tumour of lymphatic or vascular endothelial cell origin [1]. It is a highly aggressive malignant tumour with poor prognosis and accounts for less than 1–2 % of soft tissue sarcomas [[1], [2], [3], [4], [5], [6], [7]]. Breast AS can be classified as primary and secondary, arising from the consequence of previous radiotherapy or chronic lymphoedema [3,4,8]. Breast primary angiosarcoma (PAS) is a rare entity amongst breast malignancies accounting for 0.04 % of presentations [3,6,[8], [9], [10]]. It typically occurs in younger pre-menopausal women in their third to fourth decade, however still few reported cases in post-menopausal women occur [4,6,[8], [9], [10]]. Breast PAS usually presents as a rapidly enlarging and painless lump with the potential for discoloration of the overlying skin [8]. The aetiology of breast PAS remains unclear with unknown risk factors, unlike secondary breast AS, however it should remain a differential diagnosis with any rapidly enlarging breast lumps at any age. We present a rare case of a primary breast angiosarcoma in a 75-year-old female. This case is reported in line with the SCARE criteria and informed consent was obtained from patient for publication of case report [11].

## 2 Presentation of case

**Fig. 1:**
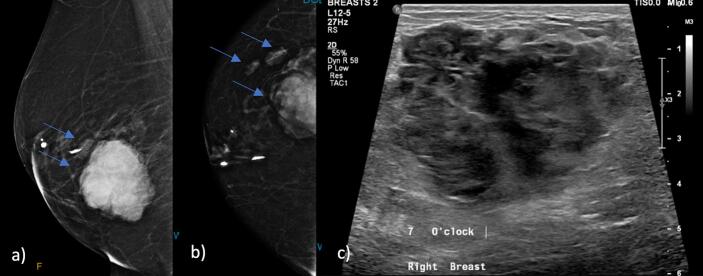
Right breast Mammogram a) Medial Lateral Oblique (MLO) projection and b) cranial caudal (CC) projection showing multiple breast lesions (blue arrow). Right breast ultrasound c) heterogonous mass. (For interpretation of the references to colour in this figure legend, the reader is referred to the web version of this article.)

A 75-year-old female referred by family practitioner, with a self-detected right breast lump. The lump was incidentally found by identification of the overlying skin discoloration with the appearance of a bruise, without a history of trauma. There were no other acute changes to shape of breast or nipple. Her past medical history includes heart failure with preserved ejection fraction, hypertension, depression, and atrial flutter, where she is on rivaroxaban. Past surgical history includes reduction mammoplasty at age 42. She is a non-smoker, minimal alcohol intake and is independent with her activities of daily living. Her family history includes breast cancer diagnosis in her sister diagnosed in her 60's, an aunt diagnosed in her 50's and a niece diagnosed in her 40's. The patient underwent triple assessment. On examination there was a large palpable and tender mass in the lower outer quadrant of the right breast with overlying ecchymosis. The contralateral breast examination revealed no abnormalities and there was no lymphadenopathy bilaterally. Breast mammogram showed large heterogenous mass in the lower outer quadrant 7 cm in diameter, and two other lesions adjacent to the primary mass measuring 1.9 cm and 6 mm in size. On ultrasound the mass was heterogenous located at 5–9 o'clock measuring 9x6x4cm. Two satellite nodules were identified which corresponded to the mammogram (Fig. 1). Initial core biopsy returned as necrotic amorphous debris with haemorrhage, likely traumatic. Due to the suspicious findings on imaging and clinical examination, repeat biopsy was organized showing haemorrhage, necrosis, fibrosis, and an area of atypical vascular proliferation with anastomosing channels lined by atypical endothelial cells. The cells were positive for CD31, CD34, ERG, and C-MYC but negative for AE1/AE3, CK7, and ER. Ki-67 shows a raised proliferation index, features favouring low-grade angiosarcoma.

Initial Tertiary Centre Sarcoma Multidisciplinary team (MDT) meeting, deemed the histology not convincing enough for angiosarcoma and suggestive of an organized haematoma due to lack of infiltrative pattern, well zonated vascular proliferation and positive MYC. They recommended wide local excision of the lesion for further diagnosis, core biopsy of satellite lesions, staging computerized tomography (CT) and PET scan.

**Fig. 2:**
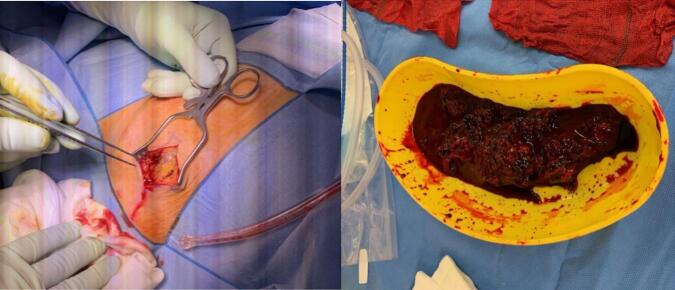
Intraoperative imaging from excisional biopsy showing large clot evacuation.

**Fig. 3:**
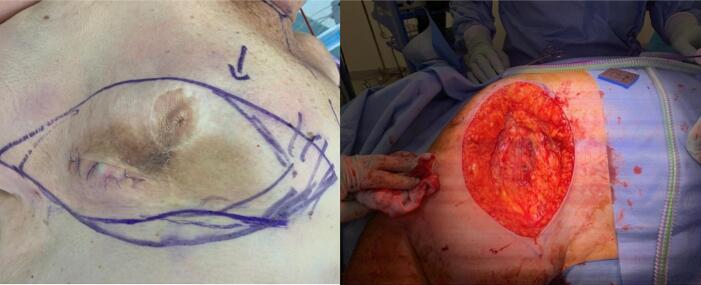
Intraoperative findings from wide local resection involving pectoralis major fascia and muscle.

On CT staging and PET scan the large mass was only mildly FDG avid and mild avidity of one of the known satellite lesions in the lateral right breast. There was no evidence of nodal or distant metastatic disease, however given the low avidity the suggestion that PET may be insensitive for diagnosis. The patient proceeded to have an excisional biopsy for further tissue diagnosis. Intraoperative findings showed a large haematoma within the inferior aspect of the right breast with associated abnormal tissue below (Fig. 2). The haematoma was evacuated, and irregular breast mass was excised with LigaSure for haemostasis. New histology confirmed moderately differentiated angiosarcoma with haematoma having some viable tumour cells present focally. A re-discussion at Sarcoma MDT suggested proceeding with further resection with wide margins, resection of pectoralis fascia, consideration of adjuvant radiotherapy, and re-discussion post-operatively. Pre-operatively the patient was reviewed by the Plastics and Reconstructive Surgery (PRS) team and a decision for a joint procedure was made. The patient underwent a right breast wide local excision, excision of pectoralis fascia and muscle underling the sarcoma and right chest wall reconstruction with pedicled latissimus dorsi (LD) flap (Fig. 3). The patient had an uneventful recovery and was discharged ten days post the operation. Histology showed moderately differentiated angiosarcoma invading into the dermis of the overlying skin and was clear of skin and soft tissue margins. A repeat PET scan was completed post operatively (six months post the initial PET) finding multiple new bilateral pulmonary nodules suggestive of metastatic disease (not PET avid). Repeat Sarcoma MDT discussion with new findings recommended discussion with medical oncology for palliative chemotherapy.

## 3 Discussion

Breast PAS is a rare form of angiosarcoma which is a mesenchymal malignant tumour [4,5]. It is associated with poor prognosis as it is described as infiltrative type of tumour resulting in high rates of metastasis and local re-occurrence [2]. Metastatic spread is thought to be hematogenous rather than lymphatic with rates of metastasis up to 50 % [3,4]. It is described as a spontaneous tumour, with few reports suggestive of association with genetic mutations like BRCA1 and BRCA2 [2,8]. Breast PAS is more commonly associated with younger women with 6–12 % diagnosed peri- or postpartum period leading to the question of hormone involvement; however there has been no established link found [7]. Most cases present with a non-tender palpable mass that is rapidly enlarging [3,7]. Radiological diagnosis is difficult, typically presenting as ill-defined masses on mammogram with no other pathognomic features in early stages of PAS [3,7,10]. Abnormal findings on mammogram suggest higher grade lesions during pathological evaluation [7]. Magnetic resonance imaging (MRI) has been suggested to be able to identify the pattern in AS of hyperintensity of mass in T2 images, rapid initial intense phase followed by washout [3,8]. As described in the case presentation, histological diagnosis through core biopsy can be difficult with similarities between AS and benign proliferation of vessels leading to a false-negative rate of 37 % [2,7]. Core biopsy can also pose problematic as tumours are usually large with areas of haemorrhage and necrosis. Most diagnoses were confirmed by excisional biopsy of the lesion or clinical course [7]. Histologically, poorly differentiated cases can also be difficult to distinguish between AS and melanoma, therefore useful immunohistochemistry markers include von Willebrand factor, CD34, CD31 *Ulex europaeus* agglutinin 1, VEGF, and melanocytic markers [[2], [3], [4]].

Current suggested optimal treatment of breast PAS is for radical surgery and adjuvant radiotherapy for local control if no evidence of metastatic disease [3,5,6,10]. Radical surgery includes mastectomy with consideration of resection of underlying muscle fascia and potentially muscle. Given the disease is primary hematogenously spread, the need for axillary dissection is unclear and not recommended in some studies [3,4,6,8]. At this stage, there is no evidence to support the utility of breast conserving surgery. Potential for limited resection if the lesion to breast ratio is appropriate for adequate margins, however, this must consider the possibility of satellite lesions [3,5]. Adjuvant radiotherapy remains undetermined if beneficial with some retrospective studies showing no difference in disease free survival (DFS) and overall survival (OS) and other studies suggesting the opposite [3]. If tumours are deemed inoperable or metastatic disease recommendations for cytotoxic chemotherapy remains mainstay and may improve disease free survival (DFS) and OS [1,3,8,10]. The role of neoadjuvant treatment is unclear [3].

## 4 Conclusion

Breast PAS is a rare entity that leads to the difficulty and delay in the diagnosis of the disease. Currently diagnostic tools including standard radiological investigations and core biopsies that can lead to false reassurance of a benign condition. Primary angiosarcoma of the breast should remain a differential in the presentation of rapidly progressing breast lumps. Consideration of early MRI scans should be discussed in those with non-specific findings on standard breast imaging associated with clinic presentation. If suspected, excisional biopsy should be considered early to help aid in diagnosis. Current management includes aggressive surgical resection however, further research needs to determine the role for neoadjuvant and adjuvant therapies. Ultimately, these patients should have appropriate discussion at MDT with specialist involvement early.

## Sources of funding

This research did not receive any specific grant from funding agencies in the public, commercial, or not-for-profit sectors.

## Ethical approval

Ethical approval not applicable.

## Consent

Written informed consent was obtained from the patient for publication of this case report and accompanying images. A copy of the written consent is available for review by the Editor-in-Chief of this journal on request.

## CRediT authorship contribution statement

Dr. Callie Killoran – study design, writing the paper.

Dr. Thushara Dissanayake - study concept/ design, editor.

## Guarantor

Dr. Callie Killoran accepts full responsibility for the work and/or the conduct of the study, had access to the data, and controlled the decision to publish.

## Declaration of competing interest

N/A.

## References

[1] Cao J., Wang J., He C., Fang M. (2019). Angiosarcoma: a review of diagnosis and current treatment. Am. J. Cancer Res..

[2] Spiker A.M., Mangla A., Ramsey M.L. (2023). https://www.ncbi.nlm.nih.gov/books/NBK441983/.

[3] Arora T.K., Terracina K.P., Soong J., Idowu O., Takabe K. (2014). Primary and secondary angiosarcoma of the breast. Gland Surg..

[4] Kunkiel M., Maczkiewicz M., Jagietto-Gruszfeld A., Nowecki Z. (2018). Primary agiosarcoma of the breast – series of 11 consecutive cases – a single-centre experience. Curr. Oncol..

[5] Sasahara A., Tanabe M., Hayashi K., Konishi T., Oya M., Sakiyama K., Morizono A., Harada M., Otsuji K., Ishibashi Y., Sato A., Kikuchi Y., Niwa T., Hinata M., Nishioka K., Seto Y. (2019). A case of primary breast angiosarcoma with multiple discontinuous small lesions. Surg. Case Rep..

[6] Darre T., Dijwa T., N’Timon B., Simgban P., Tchaou M., Napo-Koura G. (2022). Breast primary angiosarcoma: a clinicopathological and imaging study of a series cases. Breast Cancer Basic Clin. Res..

[7] Bennani A., Chbani L., Lamchahab M., Wahbi M., Alaoui F.F., Badioui I., Melhouf M.A., Amarti A. (2013). Primary angiosarcoma of the breast: a case report. Diagn. Pathol..

[8] Esposito E., Avino F., di Giacomo R., Donzelli I., Marone U., Melucci M., Rinaldo C., Ruffolo F., Saponara R., Siani C., Tortoriello R., Botti G., Rinaldo M., Fucito A. (2019). Angiosarcoma of the breast, the unknown—a review of the current literature. Transl. Cancer Res..

[9] Issar P., Ravindranath M., Dewangan M., Issar A.K. (2022). Primary angiosarcoma of the breast: a rare case report in postmenopausal women. Indian J. Radiol. Imaging..

[10] Glazebrook K.N., Magut M.J., Reynolds C. (2008). Angiosarcoma of the breast. Am. J. Roentgenol..

[11] Agha R.A., Franchi T., Sohrabi C., Mathew G., For the SCARE Group (2020). The SCARE 2020 Guideline: updating consensus Surgiacl CAse REport (SCARE) guidelines. Int. J. Surg..

